# Expansion and Functional Diversification of TFIIB-Like Factors in Plants

**DOI:** 10.3390/ijms22031078

**Published:** 2021-01-23

**Authors:** He Ning, Su Yang, Baofang Fan, Cheng Zhu, Zhixiang Chen

**Affiliations:** 1 College of Life Sciences, China Jiliang University, 258 Xueyuan Street, Hangzhou 310018, China; n15606513851@163.com (H.N.); yangsu@cjlu.edu.cn (S.Y.); 2 Purdue Center for Plant Biology, Department of Botany and Plant Pathology, Purdue University, 915 W. State Street, West Lafayette, IN 47907-2054, USA; bfan@purdue.edu

**Keywords:** transcription, transcription factors, RNA polymerases, TFIIB, plant reproduction, pollen growth, endosperm development, embryogenesis

## Abstract

As sessile organisms, plants have evolved unique patterns of growth and development, elaborate metabolism and special perception and signaling mechanisms to environmental cues. Likewise, plants have complex and highly special programs for transcriptional control of gene expression. A case study for the special transcription control in plants is the expansion of general transcription factors, particularly the family of Transcription Factor IIB (TFIIB)-like factors with 15 members in Arabidopsis. For more than a decade, molecular and genetic analysis has revealed important functions of these TFIIB-like factors in specific biological processes including gametogenesis, pollen tube growth guidance, embryogenesis, endosperm development, and plant-microbe interactions. The redundant, specialized, and diversified roles of these TFIIB-like factors challenge the traditional definition of general transcription factors established in other eukaryotes. In this review, we discuss general transcription factors in plants with a focus on the expansion and functional analysis of plant TFIIB-like proteins to highlight unique aspects of plant transcription programs that can be highly valuable for understanding the molecular basis of plant growth, development and responses to stress conditions.

## 1. Introduction

The set of genes that are expressed defines the cell. Transcription is the most important step in gene expression [1]. In eukaryotes, three multi-subunit RNA polymerase enzymes, Pol I, II, and III, transcribe the nuclear genome [2]. Pol I, II, and III synthesize the 25S ribosomal RNA (rRNA), messenger RNA (mRNA), and small untranslated RNAs including transfer RNAs (tRNA), and 5S rRNA, respectively [2]. Transcription initiation from promoters requires both the RNA polymerases and several general transcription factors [2]. During initiation, the general transcription factors recognize promoter elements, recruit the RNA polymerases and assist them in DNA opening and initial RNA synthesis. All three RNA polymerases require TATA-binding protein (TBP), which binds upstream of the transcription start site at all promoters. The three RNA polymerase initiation complexes contain a structurally and functionally conserved core to set out similar initiation process [2]. They also rely on some distinct general factors responsible for the differences that lead to gene class-specific functions [2].

Transcriptional regulation of genes is a vital process that allows the cell or an organism to define its identity during growth, development and response to intra- and extra-cellular signals [3]. Transcriptional regulation has been most extensively studies for Pol II-transcribed genes. Transcriptional regulation generally takes place at two interconnected levels [3]. The first level involves gene-specific transcription factors and the transcription apparatus. Gene-specific transcription factors typically regulate gene expression by binding specific *cis*-acting DNA elements of their target genes and recruiting cofactors and Pol II to the target genes. Transcription cofactors are proteins or protein complexes that contribute to activation (coactivators) and repression (corepressors) but contain no DNA-binding activity [3]. These cofactors include the Mediator complex, P3000, and general transcription factors. The Mediator complex plays an important role in integrating information from transcriptional activators, repressors, signaling pathways, and other regulators during transcription [4,5]. The second level of transcriptional regulation involves chromatin and its regulators. The basic unit of chromatin, the nucleosome, is regulated by protein complexes that mobilize the nucleosome or modify its histone components [6,7]. Adenosine triphosphate (ATP)-dependent chromatin remodeling complexes of the SWItch/Sucrose Non-Fermentable(SWI/SNF) family, for example, can be recruited to activate genes by mobilize nucleosomes to facilitate access of the transcription apparatus and its regulator to DNA [8]. Gene-specific transcription factors and the transcription apparatus can also recruit an array of histone-modifying enzymes to acetylate, methylate, ubiquitinate, and otherwise chemically modify nucleosomes across active genes [3]. These modifications provide surfaces for interaction with protein complexes that contribute to transcriptional control [3]. Chromatin modification is highly dynamic as enzymes that remove these modifications are also typically recruited or present at the active genes as transcription goes through the various steps of initiation and elongation [3].

There are thousands of gene-specific transcription factors in a typical eukaryotic organism that determine the tissue- and cell-specificity of gene expression [3]. By contrast, general transcription factors are required for transcription of all active genes and therefore their numbers are very limited [2]. Transcription Factor IIB (TFIIB) is a general transcription factor of Pol II and in most eukaryotes has only two homologs, Rrn7 and Brf for Pol I and Pol III, respectively [2]. Interestingly, the number of TFIIB-like proteins has expanded in plants [9]. Genetic studies have shown that mutants for Arabidopsis TFIIB-like factors are defective in pollen, endosperm, and embryo development, indicating that they are regulators of plant reproductive processes. Therefore, the so-called general transcription factors may have specialized or diversified functions in plants. In this review, we will first briefly discuss general transcription factors in eukaryotes and then provide an in-depth discussion of the expansion and functional analysis of plant TFIIB-like proteins to illustrate unique aspects of plant transcription programs that could be of high value for understanding the molecular basis of plant growth, development and responses to stress conditions.

## 2. Expansion of TFIIB-Like Factors in Plants

In eukaryotes, transcription of protein-coding genes is performed by Pol II, which utilizes up to seven different general transcription factors (TATA box-binding protein or TBP, TFIIA, TFIIB, TFIID, TFIIE, TFIIF and TFIIH) for promoter recognition and transcription initiation [1,2]. TFIIB and TBP are the best studied general transcription factors among eukaryotic transcription initiation factors. TFIIB-like factors Rrn7/MEE12 and Brf1 are required for eukaryotic Pol I and III, respectively. TBP is also universally required for transcription initiation by the three major eukaryotic RNA polymerases [2]. TFIIB-like proteins contain three domains with a zinc ribbon domain at the N-terminus followed by a linker domain and two cyclin fold repeats [2]. Brf- and Rrn7/TAF1B/MEE12-like proteins consist of the same three TFIIB-like domains, but also contain an extended C-terminal domain following the cyclin fold repeats [2]. TFIIB-like factors contact the RNA polymerase dock domain via their N-terminal zinc ribbon, and may all bind TBP and DNA via their two cyclin fold core domains. In the Pol II preinitiation complex, TFIIB simultaneously interacts with Pol II, TBP, and DNA upstream and downstream of TBP for polymerase recruitment to the promoter, forming a bridge between the promoter and Pol II. TFIIB also plays roles in transcription start site selection, promoter opening, abortive initiation, promoter clearance, and termination [2].

**Figure 1:**
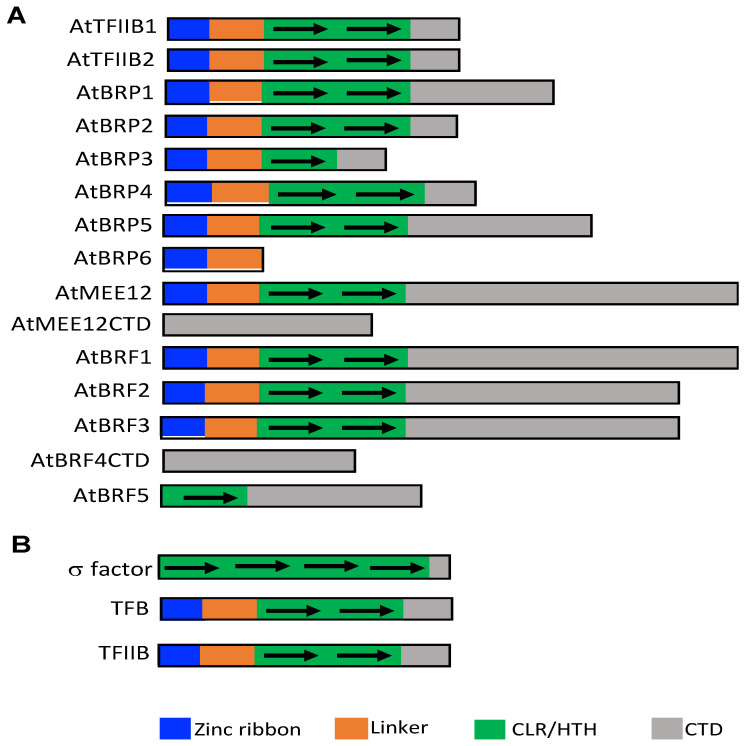
Functional domains of Arabidopsis TFIIB-like proteins in Arabidopsis (**A**) and comparison of bacterial s factor, archaeal TFB and eukaryotic TFIIB protein structures (**B**).

Unlike gene-specific transcription factors, many of which are encoded by large gene families, the families of general transcription factors are usually very small with 2–4 members [2]. In plants, interestingly, some of their general transcription factor families have expanded significantly in number. In Arabidopsis, for example, there are two genes encoding TBP [10]. In non-plant eukaryotes including yeast and human, there are usually 3 or 4 TFIIB-related proteins for Pol I, II, and III. In yeast, beside TFIIB for Pol II, there are a TFIIB paralog Rrn7 for Pol I and another paralog Brf-1 for Pol III [9]. Intriguingly, plants contain a large family of TFIIB-like proteins, raising the possibility of redundant, specialized and diversified functions of this particular group of general transcription factors [9]. In Arabidopsis, 14 TFIIB-like factors have been previously identified [9]. We have searched the more recently annotated version of the Arabidopsis genome and discovered an additional TFIIB-like factor (AtBRF5) in the model plant (Figure 1A). Among the 15 TFIIB-like proteins in Arabidopsis, 10 contain all the three domains found in TFIIB, Brf1 and Rrn7/MEE12 (a zinc domain, a linker and two cyclin fold repeats) [9] (Figure 1A). Arabidopsis TFIIB-related Protein 3 (AtBRP3) is similar to TFIIB by containing a zinc ribbon and linker domain, but only contains a single cyclin fold. The domain architecture of AtBRP6 only consists of a zinc ribbon and linker domain without the entire cyclin fold repeat domain (Figure 1A). The newly identified AtBRF5 contains the linker and one cyclin fold repeat but lacks the N-terminal zinc domain (Figure 1A). The two remaining TFIIB-like proteins, AtBRF4CTD and AtMEE12CTD, contain only sequences similar to the extended C-terminal domains of Brf1 and Rrn7/MEE12, respectively, but are missing the entire three other domains found in TFIIB-like proteins [9] (Figure 1A).

Comprehensive search and analysis of TFIIB-like proteins from different eukaryotic genomes including metazoan, fungi and algae species have confirmed that there are in general three or more TFB-like proteins in each genome [9]. However, there is a clear expansion in the number of TFIIB-like proteins in plants beginning with lycophtyes and continuing with the higher plant species [9]. The phylogenetic tree divided the TFIIB-like proteins into five distinct subfamilies [9]. In addition to the three expected TFIIB, Rrn7/TAF1B/MEE12, and Brf clades, there are two additional clades that represent the Brp1-like and Brp5-like proteins, which are only found in plant and algae species [9]. The Brp1 subfamily first emerges in red algae, is missing in green algae and Bryophyte mosses, but then clearly reemerges in the genomes of Lycophyte mosses and higher plants [9]. It is not clear why members of the Brp1 subfamily would be missing in green algae and Bryophyte mosses. The Brp5 subfamily first emerges in green algae and is continuously found in mosses and higher plant species [9]. The expansion of TFIIB-like proteins in plants raises important questions not only about the roles of these putative transcription factors but also about the general transcription program in plants as opposed to those from other eukaryotic organisms.

## 3. Eukaryotic TFIIB, Bacterial σ Factors, and Archaeal TFBs

The expansion of TFIIB-like proteins in plants is intriguing but not completely surprising given their structural, functional and evolutionary relationship with bacterial σ factors and archaeal transcription factor B (TFB). Multi-subunit DNA-dependent RNA polymerase proteins for gene transcription are conserved in bacteria, archaea, and eukaryotes. Bacterial RNA polymerase holoenzyme consists of the RNA polymerase core enzyme and a σ factor [11,12]. On binding to the RNA polymerase, bacterial σ factors allow efficient promoter recognition and transcription initiation. It is the σ factor that determines which bacterial genes to be transcribed [11,12]. Archaeal RNA polymerase is more complex with 14 subunits and utilize TFB-TBP rather than σ factors for recognition of target genes and transcription initiation [11,12]. Structural and evolutionary analysis has revealed that bacterial σ factors, archaeal TFB and eukaryotic TFIIB are homologs [11]. TFIIB and TFB each have two five-helix cyclin-like repeats (CLR) that include a C-terminal helix-turn-helix (HTH) motif (CLR/HTH domains) [11] (Figure 1B). A total of four homologous HTH motifs are also present in bacterial σ factors that are relics of CLR/HTH domains [11] (Figure 1B). Bacteria contain a primary σ factor and many alternative σ factors for coordinated expression of discrete sets of genes [13,14,15]. Archaea also have an expanded TFB protein family that potentially mediate environmental responses (e.g., heat shock and oxidative stress) of archaea [11]. In fact, the exceptional success of many archaea in environmental extremes has raised the hypothesis that expansion of the general transcription factors in these organisms might partly or fully explain their extraordinary niche adaptation capability [11].

In *Halobacterium salinarum*, a halophilic (salt-loving) member of the Archaea that grows in concentrations of NaCl near or at saturation, there are at least seven TFBs that direct environment-specific gene expression programs [16]. By correlating sequence variations, regulation, and physical interactions of all seven TFBs in *H. salinarum* NRC-1 to their fitness landscapes, functional hierarchies, and genetic interactions across 2488 experiments, covering combinatorial variations in salt, pH, temperature, and Cu stress, an elegant scheme was revealed in which completely novel fitness landscapes are generated by the introduction of subtle changes to the regulation or physical interactions of duplicated TFBs through gene conversion events [16]. These insights were used to synthetically redesigned a new TFB which, once introduced into the archaea altered the regulation of existing TFBs [16]. These results illustrate how archaea can rapidly generate novel phenotypes by simply reprogramming their TFB regulatory network.

Even in eukaryotes where the functions of general transcription factors have been discussed almost exclusively in the context of basal transcription, their possible role in the regulation of physiology may have been under-appreciated. In yeast, ethanol production could be enhanced through the mutagenesis of TFIIB, suggesting that altering the function of a general transcription factor can have significant phenotypic consequences [17]. Furthermore, several studies have unearthed a possible regulatory role for general transcription factors in cell-specific differentiation and development in eukaryotes [18,19].

## 4. Functional Analysis of Plant TFIIB-Like Factors

Although expansion of TFIIB-like factors occurs in all plants, functional analysis of the large number of these transcription factors has been reported only in Arabidopsis. Significantly, a majority of these reported studies have revealed critical roles of Arabidopsis TFIIB-like factors in reproductive processes including male and female gametogenesis pollen tube germination and growth guidance, endosperm development, and embryogenesis (Table 1).

### 4.1. AtTFIIB1 and AtTFIIB2

Arabidopsis AtTFIIB1 and AtTFIIB2 are structurally highly similar with 86% similarity in amino acid sequence and both belong to the TFIIB clade in the phylogenetic tree of TFIIB-like proteins [20]. *AtTFIIB1* is expressed in many tissues including vegetative nuclei and generative cells of pollen grains and pollen tubes, endosperm, and embryos [20]. On the other hand, *AtTFIIB2* expression is not found in the endosperm and vegetative nucleus of mature pollen [20]. In total, two transfer DNA (T-DNA) insertion mutants for *AtFTIIB1* have been isolated and analyzed for the biological functions of the TFIIB-like proteins. These mutants were drastically reduced in the genetic transmission of the *attfiib1* mutations through male gametes but did not affect female gametophytic function [20]. The mutations did not affect pollen formation but caused retarded growth of pollen tubes and affected pollen tube guidance and reception in fertilization [20]. The mutations also caused defects in endosperm development, leading to disruption of fertilization and seed development [20]. The *attfiib1* mutant plants transformed with *AtTFIIB2* driven by the *AtTFIIB1* promoter were restored in pollen tube growth, guidance, and reception completely, but only partially recovered in the seed development [20]. These results indicate that the highly similar Arabidopsis AtTFIIB1 and AtTFIIB1 may have diverged in their biological functions in part due to their distinct expression patterns [20] (Table 1).

### 4.2. AtBRP1

Arabidopsis AtBRP1 was first described almost 20 years ago as a bona fide novel plant-specific TFIIB-related protein (pBRP) encoded by a ubiquitously expressed gene and is the founding member of the BRP protein family [29]. Surprisingly, unlike other general transcription factors, AtBRP1 is primarily localized to the cytoplasmic surface of the plastid envelope and its accumulation in the nucleus was detected only after treatment of the proteasome inhibitor MG132 or in the *fus6* mutant deficient in the COP9 signalosome known to target degradation of transcription factors through proteasome-mediated process [30]. Thus, Arabidopsis AtBRP1 protein contains conditional proteolytic signals that can target these proteins for rapid turnover by the proteasome-mediated protein degradation pathway. Interestingly, under conditions of proteasome inhibition, AtBRP1 proteins accumulate in the nucleus [29]. These results suggest a possible involvement of these proteins in an intracellular signaling pathway between plastids and the nucleus in plant general transcription machinery [29].

It has been proposed that plant BRP1 is a general transcription factor for Pol I but not for Pol II based on chromatin immunoprecipitation (ChIP) assays of BRP1 from red algae *Cyanidioschyzon merolae* and Arabidopsis [21]. ChIP analysis revealed that CmpBRP1 specifically recognized the rDNA promoter region in vivo, and the occupancy was correlated to de novo 18S rRNA synthesis [21]. By contrast, no binding BRP1 was detected to the promoters of five light-responsive protein-coding genes as Pol II-dependent promoters and the promoter of 5S rDNA as Pol III-dependent promoter [21]. Furthermore, CmpBRP1 and CmTBP cooperatively bound the rDNA promoter region in vitro, and the binding site was identified at a proximal downstream region of the transcription start point [21]. The CmpBRP1 antibody severely inhibited α-Amanitin-resistant transcription from the rDNA promoter in crude cell lysate [21]. Likewise, transcription from the rDNA promoter was also inhibited when DNA template with a mutated CmpBRP1–CmTBP binding site was used [21]. CmpBRP1 was shown to co-immunoprecipitate and co-localize with the RNA polymerase I subunit, CmRPA190, in the cell [21]. Thus, together with comparative studies of Arabidopsis AtBRP1, it was concluded that BRP1 is a general transcription factor for Pol I in the cells of red algae and plants [21] (Table 1).

However, it has been reported that Arabidopsis AtBRP1 interacts with *Agrobacterium* transcription activator VirE3 and has strong effects on VirE3-depednent expression of plant protein-coding genes [22,31]. Furthermore, co-expression of VirE3 promotes accumulation of AtBRP1 in the nucleus of plant cells [22]. During *Agrobacterium tumefaciens*-mediated transformation of plant cells a part of the tumor-inducing plasmid, T-DNA, is integrated into the host genome. In addition, a number of virulence proteins are translocated into the host cell. The virulence protein VirE3 binds to the Arabidopsis AtBRP1 protein [22]. Stably expressed VirE3 in Arabidopsis under control of a tamoxifen-inducible promoter led to increased expression of 607 genes and decreased expression of 132 genes by more than three-fold [22]. One of the strongly activated genes encodes VirE2-interacting Protein 1(VIP1)-binding F-box Protein (VBF; At1G56250), a protein that may affect the levels of the VirE2 and VIP1 proteins [22]. Using Arabidopsis cell suspension protoplasts it was shown that VirE3 stimulated the VBF promoter, especially when co-expressed with AtBRP1 [22]. Although AtBRP1 is localized at the external surface of plastids, co-expression of VirE3 and AtBRP1 in Arabidopsis cell suspension protoplasts resulted in the accumulation of AtBRP1 in the nucleus [22]. These results indicate that VirE3 affects the transcriptional machinery of the host cell to favor the transformation process [22]. More importantly, AtBRP1 promotes VirE3-mediated transcription of protein-coding genes in Arabidopsis, indicating that the plant-specific BRP1 protein may also function in the regulation of genes transcribed by Pol II (Table 1).

### 4.3. AtBRP2

Arabidopsis AtBRP2 contains the two distinct domains that are characteristic of TFIIB-like factors: a conserved N-terminal zinc ribbon-containing domain and a conserved C-terminal domain with two 80-aa imperfect direct cyclin fold repeats [23]. Interestingly, close homologs of AtBRP2 are found only in the Brassicacea family [23]. Structurally, AtBRP2s are more related to TFIIB than to BRF [23]. AtBRP2 define a plant-specific TFIIB-related protein subfamily that appear to have evolved recently in members of the Brassicacea family in the history of land plants [23]. Unlike other B-factors that often display ubiquitously expression patterns, *AtPBRP2* expression is restricted to reproductive organs and seeds but not detectable in seedlings, mature leaves or roots [23]. AtBRP2 does not appear to be required for the Pol IV and V activities in siRNA-mediated chromatin silencing pathway [23]. Overall, two loss-of-function *atpbrp2* mutants were normal in growth and development. However, the mutants were significantly slower in the proliferation during the syncytial phase of endosperm development than wild-type plants [23]. During that period, the endosperm nuclei of wild-type plants undergo several rounds of nuclear divisions without cytokinesis, leading to formation of a syncytial structure with up to 250 nuclei before cellularization [23]. The *atpbrp2* mutants were normal in embryo development but had a 30% reduction in the number of endosperm nuclei [23]. Further analysis revealed that *atpbrp2* loss-of-function specifically affects the development of the syncytial endosperm, with contributions from both male and female gametophytes in endosperm proliferation [23]. The specific role of AtPBRP2 in endosperm development is furthermore supported by its ability to partially complementing the Arabidopsis *attfiib1* mutant plants [20]. AtTFIIB1 plays important roles in pollen tube growth, guidance, and reception as well as endosperm development [20] (Table 1). Expression of *AtBRP2* driven by the AtTFIIB1 promoter could rescue only the defective *attfiib1* seeds but not the other defects of the mutant [20] (Table 1).

### 4.4. AtBRP4

The protein sequence of Arabidopsis AtBRP4 is highly similar to those of AtPBRP2, AtTFIIB1 and 2, but the N-terminal zinc finger and the linker sequence are significantly longer than the three other TFIIB-like factors [24]. *AtBRP4* is expressed predominately in developing male gametophytes, primarily during anther development stages 5–9, during which time the pollen mother cells are undergoing meiosis to produce tetrads and the microspores are then released [24]. *AtBRP4* mRNA was also detected in tapetum from anther development stages 5–8. These findings strongly suggest that BRP4 may function during male gametophyte development. Unlike AtBRP2, AtTFIIB1 or 2, overexpression of AtBRP4 led to the small aerial organ phenotype [24]. On the other hand, down-regulation of expression of *AtBRP4* by a native promoter-driven RNA interference construct in Arabidopsis caused the arrest of the male gametophyte mitotic cell-cycle progression, leading to a pollen abortion phenotype with varied percentages of shrunken pollens [24]. These findings indicate that AtBRP4 is involved in the regulation of mitotic cell-cycle progression during male gametogenesis. Furthermore, the level of expression of *Origin Recognition Complex Protein 6* (*ORC6*), a cell cycle-related gene encoding a subunit of the origin recognition complex [32,33], was decreased in *AtBRP4* knockdown plants [24]. *ORC6* knockdown transgenic plants also displayed the male gametophyte defect as observed in BRP4 knockdown plants, suggesting that ORC6 acts downstream of AtBRP4 and is required for male gametophyte cell-cycle progression [24]. Taken together, these results reveal that AtBRP4 plays an important role in the regulation of mitotic cell-cycle progression during male gametogenesis, at least in part, through ORC6-regulated cell division machinery [24] (Table 1).

### 4.5. AtBRP5

*AtBRP5* was also referred to as *Pollen-expressed Transcription Factor 2* (*PTF2*) due to its expression in developing pollen grains [25]. Analysis with RT-qPCR and b-glucuronidase (GUS)- or Green Fluorescent Protein (GFP) fusion constructs further indicated that *AtBRP5* is expressed in many tissues in inflorescence, developing pollen grains and embryos [25]. Expression was also detected in shoot apical meristems, root tips, and primordia of lateral roots but not in leaves [25]. In the developing pollen grains, expression was first observed in the vegetative nuclei of the pollen grains at the early binucleate stage, persistent during the second pollen mitosis and then significantly decreased at the late trinucleate developmental stage [25]. No expression was detected in the mature pollen grains after being released from the anthers, germinating pollen grains, or pollen tubes [25]. Therefore, *AtBRP5* was expressed primarily in developing pollen grains and the tissues with active cell division and differentiation.

A mutant for *AtBRP5* with a T-DNA insertion in the 5′ untranslated region of *AtBRP5* has been identified but no homozygous mutant plants could be obtained [25]. Genetic crosses with wild-type plants indicated that transmission of *atbrp5* mutation was drastically reduced through the male gametophyte and that the mutation only had a slight impact on female gametophytic function [25]. Further analysis revealed that the mutation caused failure of pollen germination [25]. Pollen-rescue revealed that the mutation also disrupted embryogenesis and resulted in seed abortion [25]. AtBRP5 interacts with TATA-box binding protein 2 (TBP2) and bind to the double stranded DNA (dsDNA) [25]. AtBRP5 can also form a homodimer and interact with the subunits of RNA polymerases. These results strongly support that AtBRP5 plays important roles in pollen germination and embryogenesis in Arabidopsis, most likely by regulating transcription through direct interactions with TBP2 and RNA polymerases [25] (Table 1).

### 4.6. AtMEE12

AtMEE12 is a TFIIB-like factor highly similar to yeast Rrn7 and human TAF1B TFIIB-related Pol I general transcription factors. Intriguing, genetic and molecular analysis has indicated that AtMEE12 functions in the Pol II machinery to regulate pollen tube guidance [26]. RT-qPCR analysis showed that *AtMEE12* is expressed at high levels in seedlings, inflorescences, and young siliques and at lower levels in roots, but the expression is undetected in leaves and stems [26]. During reproductive development, *AtMEE12* transcripts were also expressed in flowers at different developmental stages and in siliques shortly after fertilization [26]. *AtMEE12* is also expressed in the central cell of the female gametophyte [26]. A *Ds* transposon-tagged mutant for *AtMEE12* (also known as *central cell guidance* or *ccg* mutant) is zygotically lethal, indicating an essential role of AtMEE12 in plant growth and development [26]. Analysis of the hemizygous mutant revealed that the mutation of *AtMEE2* did not affect pollen development or formation of female gametophytes but disrupted micropylar pollen tube guidance [26]. Expression of *AtMEE12* in the central cell alone is sufficient to restore the normal pollen tube guidance phenotype of the mutant [26]. These results demonstrate that the central cell plays a critical role in pollen tube guidance.

Subsequent study has shown that AtMEE12 interacts with a novel plant-specific protein called CCG BINDING PROTEIN1 (CBP1) [27]. CBP1 also interact with Mediator subunits, Pol II, and central cell-specific AGAMOUS-like transcription factors [27]. AtMEE12 interacts with Arabidopsis TBP1 and Pol II as a TFIIB-like transcription factor. *CBP1* is expressed in seedlings, leaves, inflorescences, and flowers [27]. It is also predominantly expressed in the central cells of the embryo sac [27]. Knockdown of *CBP1* expression either through a *Ds* element insertion in the 3” untranslated region or expression of artificial microRNA results in defective ovules in pollen tube attraction [27]. Expression profiling revealed AtMEE12 and CBP1 coregulate a subset of genes encoding CRPs in the central cell and the synergids, including the attractant LURE1 [27]. CRPs are small signaling peptides enriched in the embryo sac and play critical roles in pollen tube guidance, fertilization, and early embryogenesis [34,35,36,37,38,39]. These results indicate that CBP1, via interaction with AtMEE12 and the Mediator complex, links transcription factors and the Pol II machinery to regulate pollen tube attraction [27] (Table 1).

### 4.7. AtBRF1-3

AtBRF1, 2, and 3 are three closely related TFIIB-like factors highly homologous to the yeast and human BRF TFIIB-related Pol III GTFs. Expression analysis using the GUS reporter gene showed that the expression of *AtBRF1* and *AtBRF2* was mainly detected in the anthers and ovary [28]. In comparison, expression of *AtBRF2* was stronger than that of *AtBRF1*, but expression of *AtBRF3* was hardly detected [28]. qRT-PCR analysis further showed that in three-week-old seedlings grown under normal conditions the expression of *AtBRF2* was 0.7 times higher than that of *AtBRF1,* and was 1.6 times higher than that of *AtBRF3*. To directly address the biological functions of the three TFIIB-like factors, T-DNA knockout mutants were isolated but found to be normal in both growth and development [28]. To overcome the functional redundancy among the three TFIIB-like homologs, double mutants were also generated through genetic crosses. The resulting double mutants for *AtBRF1* and *AtBRF3* or for *AtBRF2* and *AtBRF3* were normal in both growth and reproduction [28]. By contrast, no double mutant for *AtBRF1* and *AtBRF2* was obtained, suggesting that a double mutation of AtBRF1 and AtBRF2 results in the infertility of Arabidopsis [28]. Further analysis found that heterozygous *atbrf1/+atbrf2/+* produced abnormal pollen and had no seeds in some placentas of siliques. Analysis using reciprocal crosses showed that AtBRF2 plays a dominant role in Arabidopsis reproduction [20]. These results indicate that mutations of both AtBRF1 and AtBRF2 lead to a high degree of aborted macrogametes and microgametes and complete failure in zygote generation [20] (Table 1).

## 5. Conclusions and Prospect

**Figure 2:**
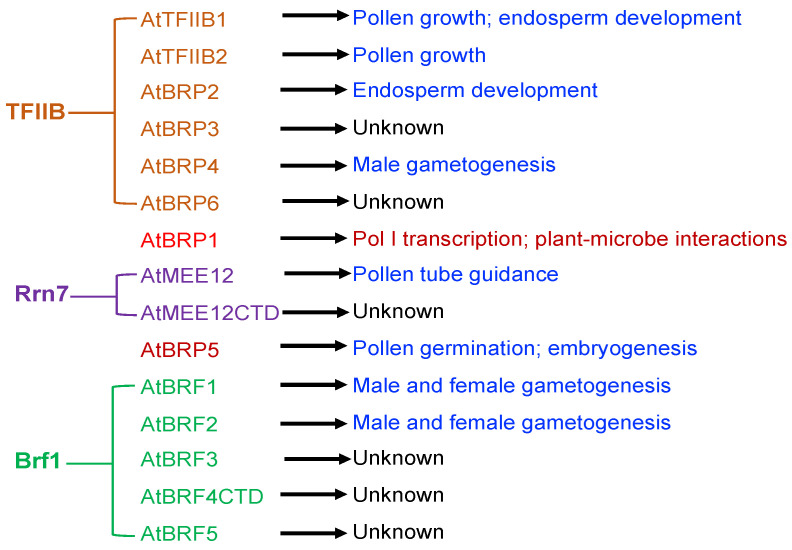
Expansion and functional diversification of Arabidopsis TFIIB-like proteins. The possible evolutionary relationship of Arabidopsis TFIIB-like factors with eukaryotic TFIIB, Rrn7 and Brf1 general transcription factors is based on the phylogenetic analysis, which has also shown Brp1-like and Brp5-like proteins to be present only in plant and algae species [9]. Molecular and genetic analysis has revealed that a majority of these Arabidopsis TFIIB-like proteins play crucial roles in plant reproductive processes.

Even though 15 TFIIB-like factors have been identified from the Arabidopsis genome, two of them (AtBRF4CTD and AtMEE12CTD) are unlikely to act as TFIIB-like factors due to the lack of the conserved N-terminal zinc finger and the two cyclin fold repeats. Among the remaining 13 TFIIB-like factors, 10 have been characterized by reported studies to various extent through molecular and genetic approaches (Table 1). Genetic and molecular analysis has established important roles of eight Arabidopsis TFIIB-like factors in specific biological processes including gametogenesis, pollen tube growth guidance, embryogenesis, endosperm development, and plant-microbe interactions (Figure 2). Establishment of so-called general transcription factors in specific biological processes is highly significant as the findings challenge the paradigm of general transcription factors as universal regulators of class-specific gene expression.

Despite these progresses, our understanding of the expanded family of plant TFIIB-like factors is still very limited. First, even though the expansion of the TFIIB-like proteins occurs in all plants, their functional analysis has so far been restricted to Arabidopsis. It is, therefore, unclear about the structural and functional conservation and diversification of the expanded TFIIB-like factors among different plants, which are diverse in a wide range of traits in growth, development and stress responses. Second, even in Arabidopsis, our knowledge about those functionally analyzed TFIIB-like factors is restricted to their structures, expression patterns and mutant phenotypes. We are still unclear about the molecular basis for the established roles of these TFIIB-like factors. In addition to the three major RNA polymerases, plants contain Pol IV and V involved in siRNA-mediated gene silencing. Some of Arabidopsis TFIIB-like factors have been demonstrated to be associated with specific RNA polymerases, but important questions remain. For example, AtMEE12 has been shown to be a transcription factor associated with Pol II in Arabidopsis despite the fact that it is structurally most closely related to human and yeast Rrn7/TAF1B factors, which act as general transcription factors for Pol I. BRP1 has been shown to be a general transcription factor for Pol I in red algae and Arabidopsis but AtBRP1 also plays a key role in the transcription of protein-coding genes regulated by the VirE3 effector from Agrobacterium. Third, understanding of the biological roles of these TFIIB-like factors will require a better knowledge about the specific transcription program directed by the TFIIB-like factors including their direct target genes. Identification of direct target genes and establishment of plant TFIIB-like factors as special transcription factors of a specific Pol will challenge the paradigm of GTFs and can potentially transform our understanding of transcriptional regulation in plants and other eukaryotes. Establishment of plant TFIIB-like factors as master regulators of important plant traits will be a breakthrough, not only for understanding transcriptional regulation of plant growth and development but also for developing novel strategies of engineering to improve crop plants. Elucidating the regulation of plant TFIIB-like factors will help understand signaling pathways for transcription reprograming in plants.

## Figures and Tables

**Table 1:** Functional analysis of Arabidopsis TFIIB-like factors.

Name/	Gene I.D.	Function	Reference
AtTFIIB1	AT2G41630	Pollen tube growth, guidance, and reception and endosperm development	[20]
AtTFIIB2	AT3G10330	Unknown, but could restore pollen tube growth, guidance, and reception of attfiib1 when driven by the AyTFIIB1 promoter.	[20]
AtBRP1	AT4G36650	Acts as a Pol I GTF, but also interacts with Agrobacterium transcription activator VirE3 and affect VirE3-depednent expression of plant protein-coding genes.	[21,22]
AtBRP2	AT3G29380	Endosperm development	[23]
AtBRP3	AT4G10680	Unknown	
AtBRP4	AT3G57370	Mitotic cell-cycle progression during male gametogenesis.	[24]
AtBRP5/TFP2	AT4G35540	Pollen germination and embryogenesis:	[25]
AtBRP6	AT5G39230	Unknown	[9]
AtMEE12/CCG	AT2G02955	Central cell-mediated pollen tube guidance.	[26,27]
AtMEE12CTD	AT4G01340	Unknown	[9]
AtBRF1	AT3G09360	Functionally redundant in both male and female gametogenesis.	[28]
AtBRF2	AT2G45100		
AtBRF3	AT2G01280	Unknown	[9]
AtBRF4CTD	AT4G19550	Unknown	[9]
AtBRF5	At1G30455	Unknown	This study

## Data Availability

No new data were created or analyzed in this study. Data sharing is not applicable to this article.
